# Side‐Gated In_2_O_3_ Nanowire Ferroelectric FETs for High‐Performance Nonvolatile Memory Applications

**DOI:** 10.1002/advs.201600078

**Published:** 2016-04-15

**Authors:** Meng Su, Zhenyu Yang, Lei Liao, Xuming Zou, Johnny C. Ho, Jingli Wang, Jianlu Wang, Weida Hu, Xiangheng Xiao, Changzhong Jiang, Chuansheng Liu, Tailiang Guo

**Affiliations:** ^1^ Department of Physics and Key Laboratory of Artificial Micro‐ and Nano‐structures of Ministry of EducationWuhan UniversityWuhan430072China; ^2^ Department of Physics and Materials ScienceCity University of Hong KongTat Chee AvenueKowloonHong Kong SARChina; ^3^ National Laboratory for Infrared PhysicsShanghai Institute of Technical PhysicsChinese Academy of SciencesShanghai200083China; ^4^ Institute of Optoelectronic DisplayFuzhou UniversityFuzhou350002China

**Keywords:** ferroelectric memory, field‐effect transistors, In_2_O_3_ nanowires, P(VDF‐TrFE), side‐gated

## Abstract

**A new type of ferroelectric FET based on the single nanowire** is demonstrated. The design of the side‐gated architecture not only simplifies the manufacturing process but also avoids any postdeposition damage to the organic ferroelectric film. The devices exhibit excellent performances for nonvolatile memory applications, and the memory hysteresis can be effectively modulated by adjusting the side‐gate geometries.

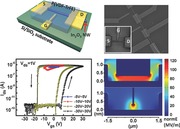

In recent years, ferroelectric field‐effect transistors (FeFETs) have been widely studied as an important type of memory due to their remarkable characteristics of nonvolatility, low power consumption, fast operation speed, and nondestructive memory operation.^1^, ^2^, ^3^, ^4^, ^5^ Until now, among all promising ferroelectric materials explored in FeFETs, organic ferroelectric polymers contain the exceptional advantages of light weight, mechanical flexibility, and low‐temperature solution‐base processing, which are ideal for the large area technological utilizations.^6^, ^7^, ^8^, ^9^, ^10^ Poly(vinylidene fluoride‐trifluoroethylene) (P(VDF‐TrFE)) copolymer is one of the typical organic ferroelectric polymers that is frequently employed in various nanoelectronic devices because of its room temperature ferroelectricity, large remnant polarization, and easy forming process with the required annealing temperature below 140 °C.^11^, ^12^, ^13^, ^14^ Importantly, the stable remnant polarization of the P(VDF‐TrFE) dielectric layer can enable an ultrahigh local electrostatic field in the semiconductor channel, which is much larger than that produced by the gate bias in traditional field‐effect transistors;^15^, ^16^ in this case, the transport properties of the channel can be effectively modulated under a relatively low gate bias or even stayed in depletion or accumulation state after the gate bias is removed. However, since P(VDF‐TrFE) is highly soluble in common organic solvents, it is cumbersome to fabricate electronic devices with P(VDF‐TrFE) in the top‐gated configuration with short channel lengths (i.e., sub‐μm) via conventional ultraviolet or electron‐beam lithography (EBL) techniques.^5^, ^17^ In addition, if the P(VDF‐TrFE) film is irradiated by the electron‐beam, the ferroelectric properties of P(VDF‐TrFE) would be degenerated into relaxor (see Figure S1 in the Supporting Information).^18^ As a result, utilizing these organic ferroelectric polymers for advanced electronics gets essentially restricted considering that the corresponding top‐gated structure needs complicated manufacturing processes while the simple back‐gated device structure is not suitable for high‐performance integrated circuits.

At the same time, 1D semiconductor nanowires (NWs) have attracted considerable amount of research attention with the detailed exploration as device channels for many different applications owing to their excellent physical properties.^19^, ^20^, ^21^, ^22^ Semiconductor NW material systems with excellent crystallinity are demonstrated with the extraordinary field‐effect carrier mobility and superior control in the modulation of their electrical properties. Combining with their nanoscale dimensions, NW devices are highly desirable for the high‐density integration of computation chips, logic, and memory cells with the further reduction in power consumption; actually, the fin field‐effect transistor which has been widely used in today's advanced integrated circuits is fabricated based on Si nanowires.^23^ Meanwhile, metal‐oxide NWs such as In_2_O_3_ is a noteworthy NW material system here because of its high mobility (≈1490 cm^2^ V^−1^ s^−1^), inexpensive synthesis, air stability, wide band gap (≈3.6 eV), and good metal–semiconductor contact.^24^, ^25^, ^26^ All these unique characteristics have made In_2_O_3_ NWs being appropriate for the efficient deployment in electronics, optoelectronics, sensors, and especially as the transistor channel for high‐performance nonvolatile memory operations.

In this work, we have investigated FeFETs using the In_2_O_3_ NW and organic P(VDF‐TrFE) copolymer with a side‐gated architecture fabricated through a relatively simple manufacturing process. The source, drain, and gate patterns were defined by only one electron‐beam lithography (EBL) step whereas the P(VDF‐TrFE) dielectric film was spin‐coated onto the sample substrate afterward. This way, there was not any lift‐off or etching process required after the deposition of the organic ferroelectric film, not only simplifying the manufacturing process but also avoiding any postdeposition damage to the organic film. In addition, the P(VDF‐TrFE) polymer film can act as a protective layer since not any other unit in the fabrication exceeds its capsulation. It is also noted that our side‐gated devices exhibit excellent performances at room temperature and in ambient air. The leakage current, on/off current ratio, and subthreshold slope (*SS*) all present superior values as compared with those of NW‐based FeFETs reported previously.^5^, ^12^, ^13^, ^17^, ^27^ Also, the memory hysteresis characteristics can be effectively controlled by adjusting the side‐gated geometrical parameters. Moreover, in order to illustrate the versatility of our newly developed NW devices, a memory inverter circuit is as well constructed, which further indicates the technological potency of our ferroelectric side‐gated structures for integrated memory circuit applications.

Specifically, crystalline In_2_O_3_ NWs with the diameters of 40–60 nm and the lengths of tens of micrometers were grown by a chemical vapor deposition (CVD) method, and transferred onto Si substrates with the 270 nm thick thermally overgrown SiO_2_ layer. **Figure** **1**a then displays the device structure of a side‐gated FeFET as a nonvolatile memory cell. Scanning electron microscopic (SEM) image of the device is shown in Figure 1b. The source, drain, and gate patterns were defined by using one EBL step, and Cr/Au (10/50 nm) metal stacks were deposited as electrodes followed by a lift‐off process. Finally, the P(VDF‐TrFE) (70:30 in mol%) film was spin‐coated onto the devices with a subsequent thermal treatment on a hot plate for improving the crystallization of P(VDF‐TrFE) ferroelectric domains. The thickness of the P(VDF‐TrFE) layer was measured to be 100 nm. During the device operation, once a suitable negative gate pulse is applied onto the gate, the polarizations of the ferroelectric dielectric layer will be aligned from the NW to the tip of the gate electrode. When the gate voltage pulse is removed, the remnant polarization of the ferroelectrics provides a local negative field effect,^2^, ^16^ which depletes the electrons in the In_2_O_3_ NW channel and results in a low drain current (i.e., the high resistance state as the off‐state for erase), as depicted in Figure 1c. In contrast, as seen in Figure 1d, when a positive *V*_gs_ pulse is applied, the NW channel becomes conductive due to the electron accumulation and allows a high drain current (i.e., the low resistance state as the on‐state for program).

**Figure 1:**
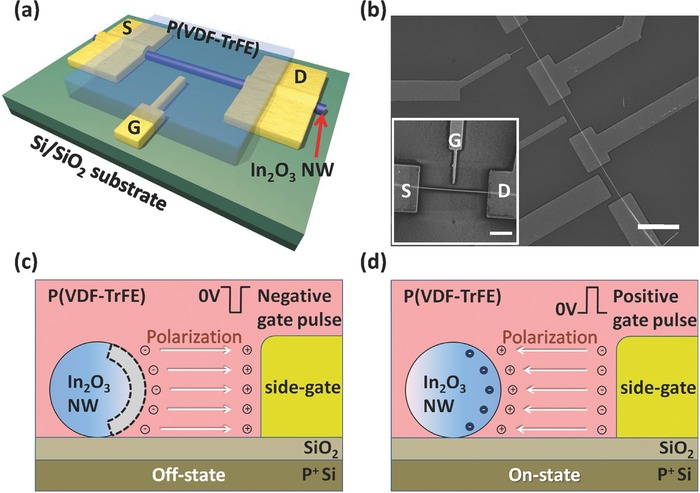
Device structure and operation schematics of a side‐gated In_2_O_3_ NW FeFET. a) Illustrative schematic of the device configuration. b) SEM image of three devices with different side‐gate widths of 100 nm, 600 nm, and 2 μm. Scale bar is 3 μm. Inset shows the structure of a single device with the channel length of 3 μm. Scale bar is 1 μm. c,d) The cross‐sectional structures of the device operating in two different working states: electrons in the NW channel get depleted after a negative gate pulse (off‐state for erase) while accumulated after a positive gate pulse (on‐state for program).

We first evaluate the transfer characteristics of fabricated devices before coating the P(VDF‐TrFE) film. As shown in **Figure** **2**a, when the device is designed with the side‐gate width (*W*) of 600 nm and NW‐to‐gate distance (*D*) of 400 nm, the electrostatic field produced by gate bias, even up to the *V*_gs_ sweep range of ±30 V, is not sufficient to deplete electrons in the NW channel. The inset of Figure 2a gives a SEM image illustrating the geometrical parameters utilized and varied in this study. After the FeFET fabrication has been completed with the final step of P(VDF‐TrFE) deposition, the transfer curve exhibits an obvious memory window for the *V*_gs_ sweep range raised to ±20 V, with the drain bias of 1 V and device channel length of 3 μm (Figure 2b). Notably, the hysteresis loop of our devices is traversed in a counterclockwise direction, which is opposite to the typical clockwise hysteresis of In_2_O_3_ NW back‐gated FETs (see Figure S2 in the Supporting Information) while keeping with the polarization hysteresis of P(VDF‐TrFE) (see Figure S3 in the Supporting Information). The form of the counterclockwise hysteresis can be understood as follows. The In_2_O_3_ nanowire used in this work is a kind of n‐type depletion semiconductor. The polarizations of the ferroelectric dielectric is aligned from the NW to the tip of the gate electrode as the negative gate bias increases upon its coercive voltage, and provides a rather high negative field to deplete carriers in the semiconductor channel. As a result, the drain current changes from on‐state to off‐state at near −20 V. As *V*_gs_ sweeping back, the ferroelectric remains polarized and the off‐state remains stable. Then the polarizations of the ferroelectric dielectric starts to change sign with the positive gate bias increases and the drain current changes from off‐state to on‐state. Due to the positive field caused by remained ferroelectric polarizations, the on‐state can also remain stable until *V*_gs_ become negative again.^28^, ^29^ The counterclockwise hysteresis demonstrates that the polarization of P(VDF‐TrFE) film has a strong effect on the transfer characteristics of our devices. The polarization characteristics of the ferroelectric film are also found to vary significantly depending on the voltage sweep rate.^30^ Therefore, all the devices are measured under the same gate voltage sweep rate of 4 V s^−1^ in order to establish a consistent platform for the subsequent comparison study among different devices.

**Figure 2:**
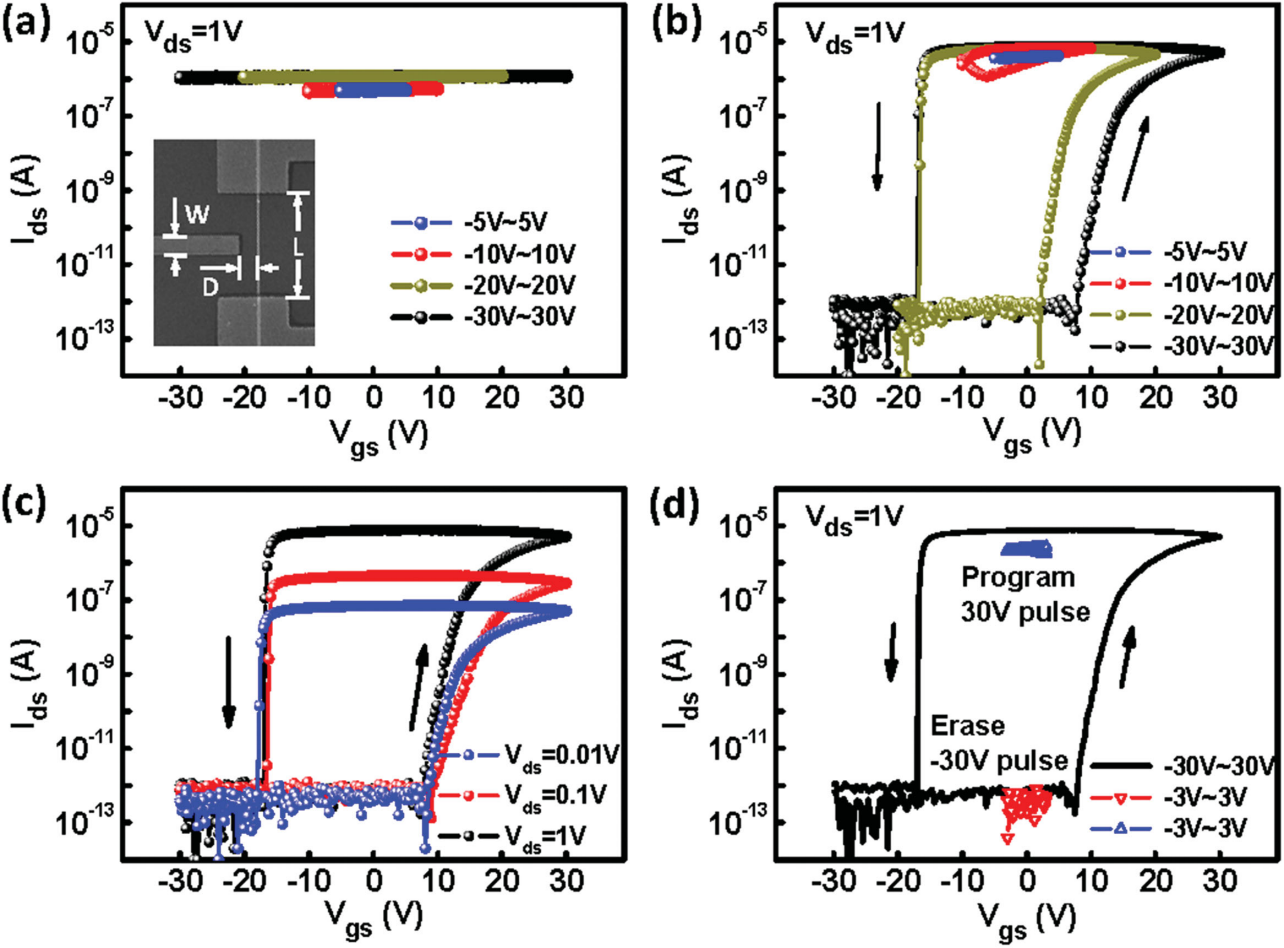
a) Transfer curves of the device before spin‐coating P(VDF‐TrFE) film, measured under different *V*_gs_ scan ranges at *V*_ds_ = 1 V. The inset gives the SEM image illustrating the geometrical parameters: side‐gate width (*W*), gate‐to‐NW distance (*D*), and channel length (*L*). b) Transfer curves of the In_2_O_3_ FeFET with a 100 nm thick P(VDF‐TrFE) film, measured under different *V*_gs_ scan ranges at *V*_ds_ = 1 V and gate voltage sweep rate of 4 V s^−1^. c) Transfer curve switch different source–drain bias ranging from 10 mV to 1 V. d) Transfer curves measured under a short‐range *V*_gs_ sweep at *V*_ds_ = 1 V, obtained after the program (30 V, 1 s) and erase (−30 V, 1 s) pulses.

Due to the polarization of the ferroelectric film, conductivity of the In_2_O_3_ NW device channel can be effectively modulated, yielding an on/off current ratio exceeding 10^6^, along with an excellent *SS* (110 mV dec^−1^) when the semiconductor channel switches from the on‐state to off‐state. In addition, the maximum gate leakage current of about 10^−12^ A (see Figure S4 in the Supporting Information) is observed repeatedly in our memory devices, being much less than the ones (10^−10^ A) of top‐gate FeFETs using P(VDF‐TrFE) as gate dielectrics,^11^, ^16^, ^31^ in which this smaller leakage can be attributed to the much smaller functional area of the side‐gated structure (i.e., only the tip of the gate electrode). Furthermore, Figure 2c gives the transfer characteristics of the device with different source–drain bias ranging from 10 mV to 1 V. It is clear that the curve does not significantly shift with the increase of source–drain bias for the same *V*_gs_ sweep range, which suggests a good transistor characteristic for these side‐gated devices. The short range *V*_gs_ sweep after the program and erase pulses also yield two distinct states in our devices, indicating their reliability, even though the corresponding ratio of maximum and minimum drain currents is slightly smaller than that observed in the transfer characteristics of long range *V*_gs_ scan, as shown in Figure 2d.

In order to explore the impact of the side‐gate geometry on device performances, we fabricated a group of In_2_O_3_ NW FeFETs with different *W* and *D*. Fabricated devices with *W* of 100 nm (narrow gate) and 2 μm (wide gate) are measured, respectively, at different *D*. **Figure** **3**a,b presents the transfer characteristics of devices with *D* of 200 and 800 nm, accordingly. All these devices are measured under the sweep range of ±50 V. It is noted that for the long *D*, the transistors cannot be cut off under a low negative gate voltage but they do show larger memory windows than the devices with gate tip close to the NW (i.e., short *D*). In addition, devices with narrow gate electrode give larger memory windows than the ones of wide gate electrodes for the same *D*. To further understand and analyze all these observed phenomena with different side‐gated geometrical parameters, we perform a simulation to assess the electric field distribution in the channel region by using finite element analysis method. Figure 3c shows the distribution of the modulus of electric field strength in the horizontal plane when *W* is designed as 100 nm and 2 μm, respectively. Figure 3d demonstrates the electric field strength distribution along a line segment from the center of the nanowire to the center of the side‐gate tip at different *W* and *D*. Evidently, the electric field strength decreases with the increasing *D* under the same gate voltage, bringing about the much larger memory window for devices with long *D*. Also, both narrow and wide gate devices have dissimilar electric field distribution between the side‐gate tip and NW, which concretely shows that the narrow gate electrode generates a spatially decayed field while the wide gate electrode induces a relatively uniform field. The nucleation and growth of switched domains follow different processes under the uniform versus decayed field; in this case, polarization switching occurred in low‐field region of decayed field requires much more time consumption.^30^ As a result, devices with narrow gate electrode show larger memory windows than the ones with wide gate electrode.

**Figure 3:**
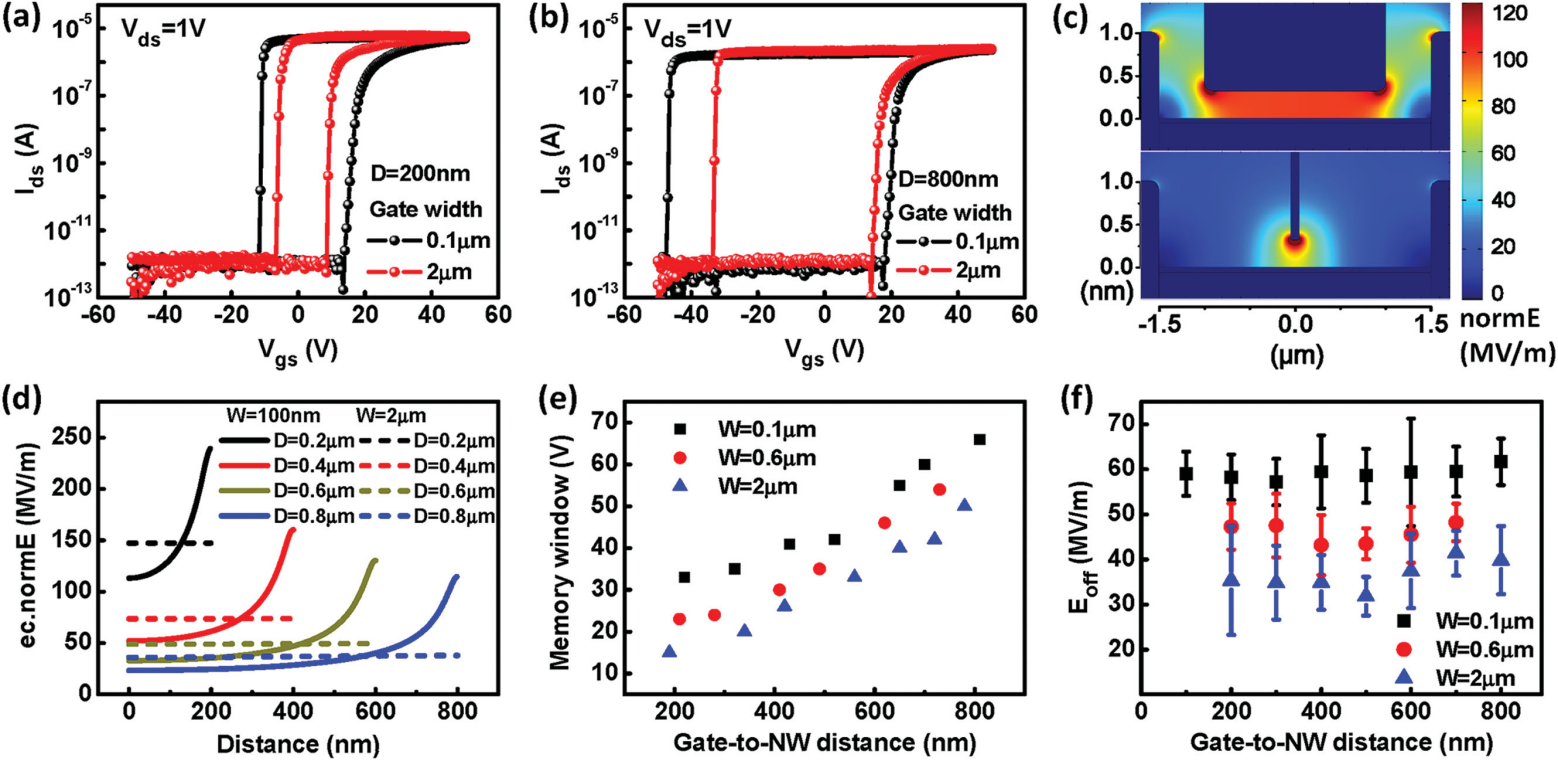
a,b) Transfer curves of the devices with different side‐gate geometries. c) Electric field distribution in the horizontal plane under *V*_gs_ = 50 V. The upper one shows the field distribution of the device with *D* = 300 nm and *W* = 2 μm while the lower one shows the field distribution of device with *D* = 300 nm and *W* = 100 nm. d) Electric field distribution in the horizontal plane along a segment from the center of the NW to the center of the side‐gate tip. e) Geometrical dependence of the memory window width. f) Average values of *E*_off_ of devices with different geometrical parameters.

We then further focused on the devices with uniform *W* but varied *D*. Figure 3e depicts the size of memory windows of these devices divided by their corresponding *W*. Geometrical parameters are measured by SEM after the electrical characterization and the measured values are all rounded to the nearest 10 nm. It is clear that the changes in memory windows are consistent with the observation discussed above. Moreover, in view of the performance variation accompanied with the nonuniformity of In_2_O_3_ NW thickness as well as process deviation in the device fabrication, a statistical study of electrical properties of fabricated devices is necessary in order to obtain a better understanding and to confirm the geometrical effect here. The turn‐off voltage (*V*_off_, defined as the gate voltage at which *I*_ds_ reaches its minimum when *V*_gs_ sweeps from +50 to −50 V in this study) is chosen to reveal the polarization switching induced by the P(VDF‐TrFE) film because the turn‐on voltage (*V*_on_, defined as the gate voltage at which *I*_ds_ starts to increase when *V*_gs_ sweeps from −50 to +50 V) is always less stable as compared with *V*_off_ and the switching from the on‐state to the off‐state is relatively sharp (see Figure S5 in the Supporting Information).^15^, ^32^, ^33^ Particularly, we compile a statistics regarding the value of *E*_off_, where *E*_off_ refers to the turn‐off electric field and it can be simply calculated by *V*_off_/*D*. Figure 3f shows the *E*_off_ of devices with different geometrical parameters of *W* and *D*, in which all these values are measured by SEM after the electrical characterization and rounded to the nearest 100 nm. More than five devices are measured for each data in the graph. For a fixed *W*, the *E*_off_ of a device does not change dramatically with various *D*, fitting well into the thickness dependence model of coercive field of the polyvinylidene fluoride films reported in previous studies,^34^, ^35^ considering that the thickness of P(VDF‐TrFE) between the gate tip and the NW changes from 200 to 800 nm. In addition, the *E*_off_ turns smaller with the increasing *W*, which is in good agreement with the above‐discussed simulation results. It is as well worth mentioning that the coercive electric field of P(VDF‐TrFE) film appears to be around 50 MV m^−1^ at the sweep loop frequency of less than 0.1 Hz,^30^ being larger than the turn‐off electric field of devices with *W* of 2 μm. We attribute this to our devices that they are operated under the interaction of ferroelectric polarizations and charge traps existed at the interface between the ferroelectric film and the NW;^28^, ^33^, ^36^ therefore, a lower electric field is required. According to the findings of these geometrical effects, we can then optimize the device design and fabrication for different utilizations with varied operating voltages.

After that, we next test the memory behavior of our In_2_O_3_ NW FeFETs. Based on the geometrical effect observed above, devices with *D* of 400 nm and *W* of 600 nm are emphasized with the program voltage of 30 V and erase voltage of −30 V in order to assess their device stability and endurance. As shown in **Figure** **4**a, both on‐ and off‐state are found to be retained stably for at least 50 000 s with an on/off current ratio of about 10^6^. Moreover, as given in Figure 4b, the stabilized program and erase cycle endurance is also observed beyond 400 cycles. The current dynamic characteristic of the memory cell is shown in Figure 4c. The device is programmed and erased at *V*_gs_ of 30 and −30 V with a 0.3 s pulse, respectively, and is read at a *V*_gs_ of 0 V, as shown in Figure 4c upper panel; the corresponding *I*_ds_ in the same time range is given in the lower panel. Although the gate pulses used in the dynamic characteristic test are much shorter than those used in stability and endurance tests, a dynamic retention ratio of about 10^5^ is still observed under *V*_ds_ of 0.1 V. Here we also studied the current dynamics of devices with the same *D* and different *W* (see Figure S6 in the Supporting Information), the result is in good agreement with our discussion about the impact of different gate sizes. All these results demonstrate an excellent nonvolatility memory behavior of our side‐gated FeFETs, by reference to ferroelectric memory with top‐gated or back‐gated structure in previous works.^5^, ^13^, ^17^, ^28^, ^36^

**Figure 4:**
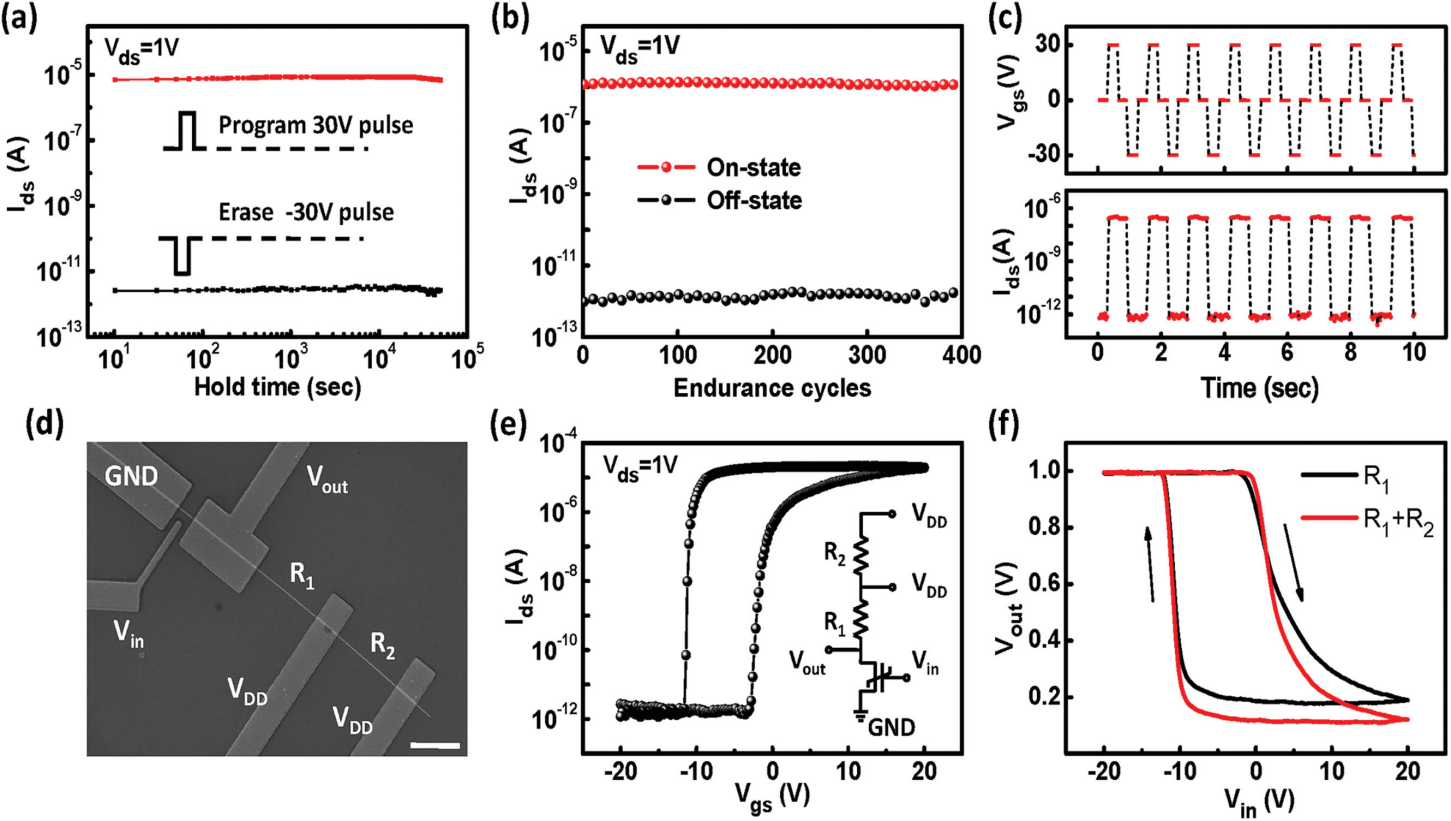
a) Retention characteristics of both on‐ and off‐state up to 5 × 10^4^ s with the duration of program and erase gate bias of 1 s. b) Program and erase endurance cycles of the device. *V*_gs_ = 0 V, *V*_ds_ = 1 V. For clarity, one cycle out of every 10 is plotted. c) Current dynamics of the memory cell, *V*_ds_ = 0.1 V. d) SEM image of the memory inverter circuit. Scale bar is 2 μm. e) Transfer characteristics of the transistor constructed in our inverter circuit. Inset shows the equivalent circuit diagram of the inverter circuit. f) VHCs of the memory inverters with different resistor types R_1_ and (R_1_+R_2_), obtained at *V*_DD_ = 1 V.

Finally, we fabricated a memory inverter circuit based on a single NW in order to investigate the feasibility of applying our side‐gated FeFETs to construct integrated memory circuits. Figure 4d depicts the SEM image of our memory circuit, which is composed of a side‐gated NW FeFET and two NW‐based resistors. The equivalent circuit diagram is displayed in Figure 4e inset. It is noted that the channel length of the NW FeFET is designed to be 800 nm to enlarge the on‐state conductance, resulting in the transfer curve shown in Figure 4e. In brief, two types of memory inverters are tested with their respective resistors R_1_ and (R_1_+R_2_). As shown in the voltage hysteresis characteristics (VHCs) in Figure 4f, our memory circuit can output a voltage representing the opposite logic level to its input with a hysteresis window approximately equal to the one of the NW‐FeFET in the circuit. The inverter with resistors (R_1_+R_2_) has a smaller low‐level output voltage owing to its larger resistance. The short‐range *V*_gs_ sweep after the program and erase pulses can also confirm the memory effect of our inverter circuit (see Figure S7 in the Supporting Information). Furthermore, the distinguishability of disparate states in the memory circuit can be estimated by the memory output ratio,^5^ which is defined as (*V*_out,Pro_ − *V*_out,Er_)/*V*_DD_ × 100%. With resistors (R_1_+R_2_), a memory output ratio of ≈85% is achieved in the inverter. As a result, all these have further demonstrated that our side‐gated FeFETs are highly compatible with integrated memory circuits in practice as they can be tuned individually and operated independently, acting like top‐gated devices.

In summary, FeFETs using the single In_2_O_3_ NW and organic P(VDF‐TrFE) copolymer dielectric with a side‐gated architecture have been demonstrated. There is not any other process required after the deposition of the organic ferroelectric film, not only simplifying the manufacturing process but also avoiding any postdeposition damage to the organic film. The fabricated devices exhibit excellent performances such as the large on/off ratio (>10^6^), small *SS* (110 mV dec^−1^) and insignificant leakage current (<10^−12^ A). The impact of this side‐gate geometry on the device performance has also been discussed. The memory hysteresis window and the state switching voltage would get larger when the side gate separates farther away from the channel and the gate electrode becomes thinner. During the typical memory operation with the program and erase pulses of ±30 V, the devices with optimized geometries yield a stable retention for over 5 × 10^4^ s, long endurance beyond 400 cycles, indicating their nonvolatility and memory performance. At the end, a memory inverter circuit is fabricated, which confirms that our side‐gated FeFETs are highly compatible with integrated memory circuits. All these results have illustrated the great potency of our ferroelectric side‐gated In_2_O_3_ NW structures for high‐performance nonvolatile memory applications.

## Experimental Section

*Nanowire Synthesis*: The single crystalline In_2_O_3_ NWs used in this study were synthesized in a horizontal tube furnace by a CVD method. In_2_O_3_ powder and graphite powder were mixed with a weight ratio of 10:1 and then put into a quartz boat. Silicon substrates with 1 nm thick of gold catalyst predeposited on the surface were placed upside‐down in the downstream position about 10 cm away from the evaporation source. Then, the entire set‐up was inserted into a quartz tube reactor. The source and substrate were heated to 1100 and 900 °C, respectively, and kept heating for 1 h under a constant flow of mixed gas (argon/oxygen = 100:1) at a flow rate of 200 sccm. When the system was cooled down to room temperature, a large amount of NWs was formed on the surface of the silicon substrates.

*Device Fabrication and Characterization*: After the growth, the In_2_O_3_ NWs were transferred onto a precleaned p‐type silicon substrate with a 270 nm thick thermally overgrown SiO_2_ layer. Then the substrates were spin‐coated with methyl methacrylate (MMA) and poly(methyl methacrylate) (PMMA), and the EBL (JEOL 6510 with nanometer pattern generation system) was employed to define the source, drain, and gate pattern. The Cr/Au (10/50 nm) electrodes were completed by thermal deposition and lift‐off processes. Finally, P(VDF‐TrFE) (70:30 mol%) ferroelectric polymer powder was dissolved in the diethyl carbonate with 2.5% wt. and the P(VDF‐TrFE) film was spin‐coated onto the substrates, followed by the thermal annealing on a hot plate (70 °C for 1 h, and 130 °C for 1 h). Electrical performance of fabricated side‐gated NW FETs was assessed using a Lakeshore probe station with Agilent analyzer B1500A and B2912A.

## Supporting information

As a service to our authors and readers, this journal provides supporting information supplied by the authors. Such materials are peer reviewed and may be re‐organized for online delivery, but are not copy‐edited or typeset. Technical support issues arising from supporting information (other than missing files) should be addressed to the authors.

Supplementary
