# Supplementary material for: Side‐Gated In2O3 Nanowire Ferroelectric FETs for High‐Performance Nonvolatile Memory Applications
Source: Adv Sci (Weinh). 2016 Apr 15;3(9):1600078. doi: 10.1002/advs.201600078 (PMC5039971; doi:10.1002/advs.201600078)
Supplement: Supplementary file 1 — Supplementary [file ADVS-3-0f-s001.pdf]

Copyright WILEY-VCH Verlag GmbH & Co. KGaA, 69469 Weinheim, Germany, 2016.

ADVANCED SCIENCE
Open Access

Supporting Information

for *Adv. Sci.*, DOI: 10.1002/advs.201600078

Side-Gated In_2O_3 Nanowire Ferroelectric FETs for High-Performance Nonvolatile Memory Applications

Meng Su, Zhenyu Yang, Lei Liao, Xuming Zou, Johnny C. Ho, Jingli Wang, Jianlu Wang,* Weida Hu, Xiangheng Xiao, Changzhong Jiang, Chuansheng Liu, and Tailiang Guo*

Supporting Information

Side-gated In_2O_3 nanowire ferroelectric FETs for high-performance non-volatile memory applications

*Meng Su, Zhenyu Yang, Lei Liao *, Xuming Zou, Johnny C. Ho, Jingli Wang, Jianlu Wang *, Weida Hu, Xiangheng Xiao, Changzhong Jiang, Chuansheng Liu, and Tailiang Guo*

[*] Prof. L. Liao, Dr. M. Su, Dr. Z.Y. Yang, Dr. X.M. Zou, Dr. J.L. Wang, Prof. X.H. Xiao, Prof. C.Z. Jiang, Prof. C.S. Liu
Department of Physics and Key Laboratory of Artificial Micro- and Nano-structures of Ministry of Education, Wuhan University, Wuhan 430072,China
E-mail: liaolei@whu.edu.cn
Prof. J. C. Ho
Department of Physics and Materials Science, City University of Hong Kong, Tat Chee Avenue, Kowloon, Hong Kong SAR, China
Prof. J.L. Wang, Prof. W.D. Hu
National Laboratory for Infrared Physics, Shanghai Institute of Technical Physics, Chinese Academy of Sciences, Shanghai, 200083, China
E-mail: jlwang@mail.sitp.ac.cn
Prof. T.L. Guo
Institute of Optoelectronic Display, Fuzhou University, Fuzhou 350002, China

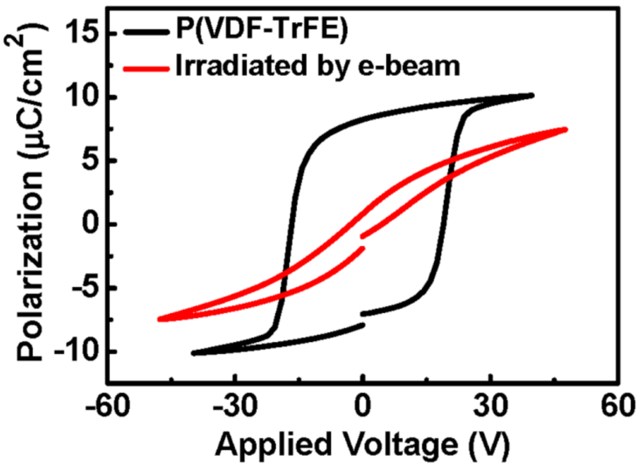

Figure S1. The relationship between polarization vs. applied voltage. Fresh P(VDF-TrFE) film (black) and the film irradiated by e-beam (red).

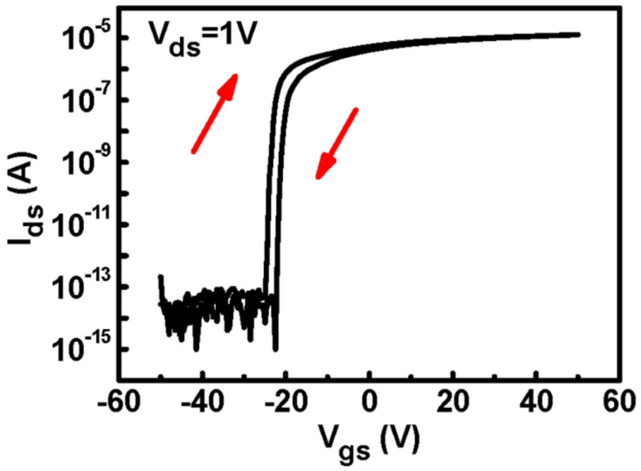

Figure S2. Transfer curve of the back-gated In_2O_3 NW FET, under V_{gs} sweep range ±50V at V_{ds} = 1 V.

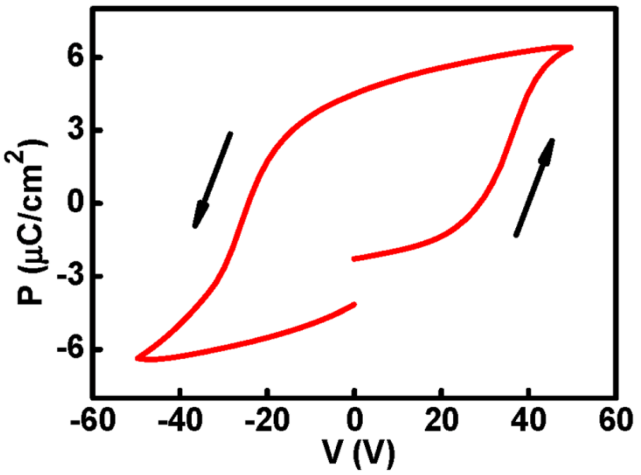

Figure S3. The ferroelectric hysteresis loop 300 nm P(VDF-TrFE) film capacitor measured at 1 kHz applied voltage frequency.

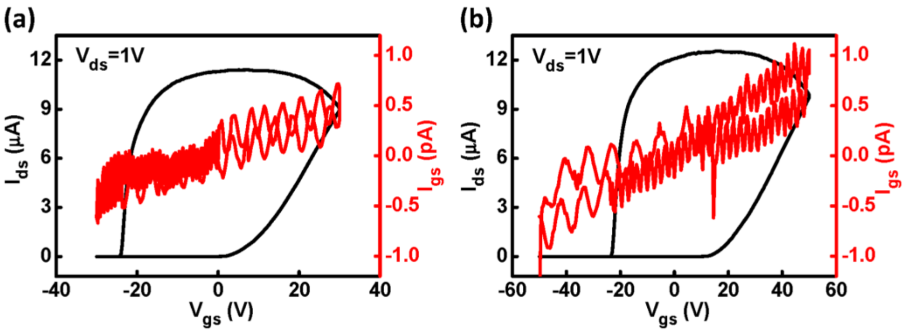

Figure S4. I_{gs}-V_{gs} dependence of the side-gated In_2O_3 NW FeFET with 600 nm side gate width and 500 nm NW-gate distance at V_{ds} = 1 V. (a) Under V_{gs} sweep range ±30 V. (b) Under V_{gs} sweep range ±50 V.

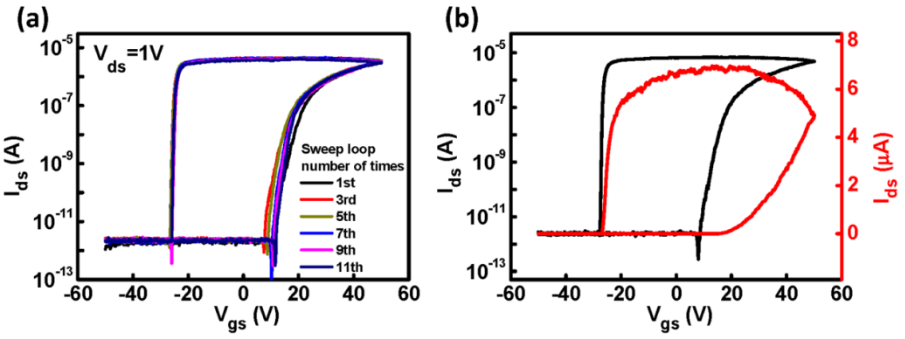

Figure S5. (a) I_{gs}-V_{gs} dependence of the side-gated In_2O_3 NW FeFET within 11 sweep loops. For clarity, one loop out of every two is plotted. (b) Transfer curve shown with log coordinate (black) and liner coordinate (red), respectively.

The turn-off voltage (V_{off}) is defined as the gate voltage at which I_{ds} reaches its minimum when V_{gs} sweeps from positive voltage to negative voltage while the turn-on voltage (V_{on}) is defined as the gate voltage at which I_{ds} reaches its minimum when V_{gs} sweeps from negative voltage to positive voltage. It is obvious that V_{off} is more stable than V_{on} with the gate voltage sweeps repeatedly. Moreover, the V_{off} is very close to the corresponding threshold voltage (V_{th}) while the V_{on} is relative far from its corresponding V_{th}.

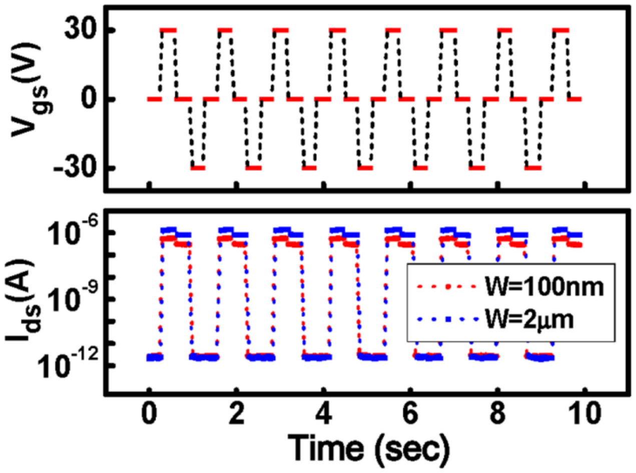

Figure S6. Current dynamics of devices with different gate widths (W), V_{ds}= 0.1 V. Both of the devices have the same D of 300 nm.

Both of the devices are programmed and erased at V_{gs} of 30 V and -30 V with a 0.3 s pulse, and are read at a V_{gs} of 0 V, as shown in the upper panel; the corresponding I_{ds} in the same time range are given in the lower panel. The drain current after the positive gate pulse is mainly determined by the degree of polarization. The device with wide gate presents larger drain current than the device with narrow gate after the same positive gate pulse, indicating that the polarization switching in the device with wide gate reaches a higher level.

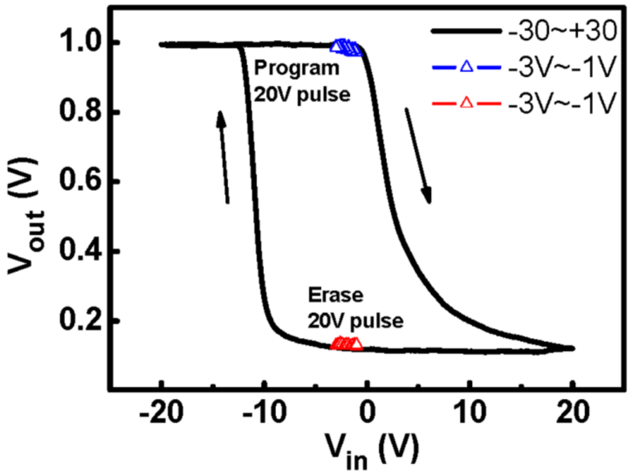

Figure S7. Short-range V_{gs} sweep (-3V~-1V) of the memory inverter circuit after the program and erase pulses.
